# Linking lifestyle and foraging strategies of marine bacteria: selfish behaviour of particle‐attached bacteria in the northern Adriatic Sea

**DOI:** 10.1111/1758-2229.13059

**Published:** 2022-03-31

**Authors:** Vincenzo Manna, Luca Zoccarato, Elisa Banchi, Carol Arnosti, Hans‐Peter Grossart, Mauro Celussi

**Affiliations:** ^1^ National Institute of Oceanography and Applied Geophysics – OGS Department of Oceanography Trieste Italy; ^2^ Leibniz Institute for Freshwater Ecology and Inland Fisheries (IGB) Department of Experimental Limnology Zur alten Fischerhuette 2, D‐16775 Stechlin Germany; ^3^ University of North Carolina – Chapel Hill Department of Earth, Marine, and Environmental Sciences Chapel Hill NC 27599 USA; ^4^ Potsdam University Institute for Biochemistry and Biology Maulbeeralle 2, D‐14469 Potsdam Germany

## Abstract

Microbe‐mediated enzymatic hydrolysis of organic matter entails the production of hydrolysate, the recovery of which may be more or less efficient. The selfish uptake mechanism, recently discovered, allows microbes to hydrolyze polysaccharides and take up large oligomers, which are then degraded in the periplasmic space. By minimizing the hydrolysate loss, selfish behaviour may be profitable for free‐living cells dwelling in a patchy substrate landscape. However, selfish uptake seems to be tailored to algal‐derived polysaccharides, abundant in organic particles, suggesting that particle‐attached microbes may use this strategy. We tracked selfish polysaccharides uptake in surface microbial communities of the northeastern Mediterranean Sea, linking the occurrence of this processing mode with microbial lifestyle. Additionally, we set up fluorescently labelled polysaccharides incubations supplying phytodetritus to investigate a ‘pioneer’ scenario for particle‐attached microbes. Under both conditions, selfish behaviour was almost exclusively carried out by particle‐attached microbes, suggesting that this mechanism may represent an advantage in the race for particle exploitation. Our findings shed light on the selfish potential of particle‐attached microbes, suggesting multifaceted foraging strategies exerted by particle colonizers.

## Introduction

Heterotrophic microbial communities process much of the oceanic organic matter (OM) pool (Carlson *et al*., 2007), transforming energy and matter into molecules essential for life (Falkowski *et al*., 2008). The OM reservoir is shaped by a myriad of input and removal processes, resulting in a continuum of chemical and physical arrangements (Verdugo *et al*., 2004; Dittmar *et al*., 2021). Heterotrophic microbes preferentially direct their degradative efforts towards the high‐molecular weight (HMW) end of this continuum, as it tends to be more bioavailable than the low‐molecular weight (LMW) counterpart (Benner and Amon, 2015). Since the direct uptake of molecules through the prokaryotic cell wall is size constrained (i.e. <600 Da; Weiss *et al*., 1991), the drawback of this food selectivity is the need to hydrolyze these HMW substrates before exploiting them. To this purpose, microbes use extracellular enzymes, which exist as cell‐bound (i.e. attached to the outer cell membrane) and cell‐free (i.e. dissolved into the surrounding environment; Chróst, 1990). By linking the enzyme‐producing strategies with microbial lifestyle, a general microbial foraging framework can be outlined. Current hypotheses indicate that microbes dwelling as free‐living (FL) would preferentially use cell‐bound exoenzymes to cope with the patchy OM distribution in the ocean, whereas particle‐attached (PA) cells, due to their proximity to the OM sources, may mainly rely on cell‐free exoenzymes (Traving *et al*., 2015; Baltar, 2018).

Whether cell‐free or cell‐bound, exoenzymes represent a considerable energetic investment (Arnosti, 2011). When producing exoenzymes, microbes operate, to a greater or lesser extent, at a loss, as a fraction of the LMW hydrolysis products will inevitably benefit cells that do not invest in exoenzyme production (Smith *et al*., 1992; Folse and Allison, 2012). This scenario delineates two macro‐categories of microbes: sharing and cheating (i.e. enzyme‐producers and non‐producers, respectively; Allison, 2005). In this framework, PA cells may be considered as the archetype of sharing microbes. Indeed, by degrading particulate organic matter (POM) through cell‐free exoenzymes, PA microbes ‘share’ hydrolysis products with other members of the degrading consortium (Vetter *et al*., 1998; Datta *et al*., 2016). The discovery in the ocean of an alternative HMW‐OM uptake mechanism, consisting in the transport of large, partially hydrolyzed, polysaccharide fragments into the periplasmic space of the cells, where further degradation occurs, challenged this two‐player view (Reintjes *et al*., 2017). Through this mechanism, HMW‐OM can be directly taken up, minimizing the loss of LMW products derived from hydrolysis through cell‐bound or cell‐free exoenzymes (Reintjes *et al*., 2017). These ‘selfish’ microbes represent a third player in microbial substrate utilization models, competing with those prokaryotes that preferentially use cell‐bound or cell‐free exoenzymes for HMW‐OM degradation (Reintjes *et al*., 2019).

Over the last few years, the selfish uptake of several polysaccharides has been shown to be widespread across marine systems, suggesting that this substrate processing mode is highly relevant for global biogeochemical dynamics (Arnosti *et al*., 2021). Indeed, the ability to utilize biogeochemically relevant polysaccharides (i.e. pullulan, xylan, laminarin, chondroitin sulfate and arabinogalactan), both through external hydrolysis and selfish uptake, is widespread among marine bacteria (Arnosti, 2011; Reintjes *et al*., 2019; Reintjes *et al*., 2020a, 2020b). A substantial fraction of surface microbial communities can perform selfish polysaccharide uptake, including members of Bacteroidetes, Planctomycetes and Gammaproteobacteria (Reintjes *et al*., 2019, 2020a,b). Representatives of these taxa are specifically involved in the degradation and remineralization of algal biomass (Grossart *et al*., 2005; Teeling *et al*., 2012) and are often found associated with particles in aquatic environments (Zoccarato and Grossart, 2019). Nonetheless, to date, investigations specifically looking for the presence of selfish bacteria on organic particles are missing. Since PA microbes process up to 70% of POM globally (Giering *et al*., 2014), assessing how this carbon pool is processed along the selfish‐sharing gradient is of prime importance to better resolve the microscale carbon fluxes that shape global biogeochemical dynamics.

To examine the occurrence of the selfish substrate processing mode among PA and FL microbes, we incubated surface microbial communities of the northeastern Mediterranean Sea with fluorescently labelled polysaccharides (FLA‐PS), covering an annual cycle (from January 2019 to January 2020) at monthly intervals. The selfish uptake mechanism allows the exploitation of HMW‐OM without the production of hydrolysis by‐products (Cuskin *et al*., 2015), which, under the current microbial foraging theory, would be an advantageous scenario for FL microbes. Therefore, we hypothesized that selfish polysaccharide uptake would be more prominent in the FL fraction of the sampled microbial community.

In addition, we performed a second set of FLA‐PS incubations, first removing larger particles and their climax assemblages, and then supplying fresh phytodetrital particles to the microbial community. With this setup, we aimed to achieve a ‘new‐colonization’ scenario, to test whether PA microbes would selfishly exploit phytodetrital particles from the beginning of the particles' colonization process or would use this uptake mode to gain a competitive advantage in later colonization stages.

## Results and discussion

### Selfish polysaccharide uptake – natural samples

To evaluate the distribution of selfish polysaccharide uptake mechanisms among the PA and FL fraction of microbial communities, surface (~1 m depth) seawater was sampled monthly from January 2019 to January 2020 at a coastal station in the northern Adriatic Sea. Subsamples were taken in 15 ml vials, spiked with one of 5 FLA‐PS (pullulan, xylan, laminarin, chondroitin sulfate and arabinogalactan) and incubated at *in situ* temperature for 2 h. Triplicate 5 ml aliquots, from each substrate incubation, were prepared for epifluorescence microscopy analysis (as detailed in the study by Celussi *et al*., 2017). Total and FLA‐PS positive cells were identified and enumerated as FL or PA at 1000× magnification (Olympus BX60F5) under a UV (BP 330–385 nm, BA 420 nm) and blue (450–480 nm, BA 515 nm) filter set for 4,6‐diamidino‐2‐phenylindole (DAPI, Sigma Aldrich) and FLA‐PS fluorescence, respectively, in at least 15 randomly selected fields. In DAPI fields, at least 100 and 200 cells were counted for PA and FL lifestyles, respectively; under blue light, between 5 and 50 cells were counted for each replicate to measure the abundance of FLA‐PS‐stained cells. Cells were identified as substrate‐stained if they showed overlapping signals in both DAPI and FLA‐PS fields. No FLA‐PS‐stained cells were observed in the killed controls, prepared as described in the Supporting Information S1. Detailed experimental procedures and representative micrographs of FLA‐PS‐stained cells (Supporting Information Fig. S1) are available in the Supporting Information S1.

**Fig. 1:**
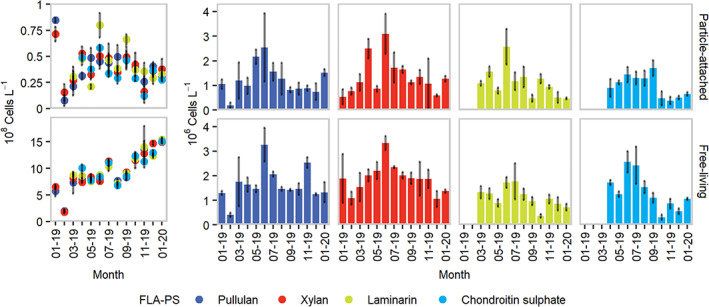
Temporal trends of total DAPI‐stained (left panel) and selfish (right panel) cell counts in unamended (i.e. without phytodetritus) samples incubated with different FLA‐Ps. Error bars depict standard deviations calculated from three experimental replicates. For visualization purposes, X‐axis labels are displayed every other month.

Through the study period (January 2019 to January 2020), microbial communities from surface water of the northern Adriatic Sea showed selfish uptake of four out of the five tested FLA‐PS (Fig. 1). Selfish uptake of pullulan and xylan was measurable throughout the study period, whereas laminarin‐ and chondroitin sulfate‐positive cells were observed starting from March and April 2019, respectively. Although some arabinogalactan‐positive cells were observed, their abundance was not high enough to statistically validate the epifluorescence counts. No substrate‐stained cells were observed in any of the killed controls.

**Fig. 2:**
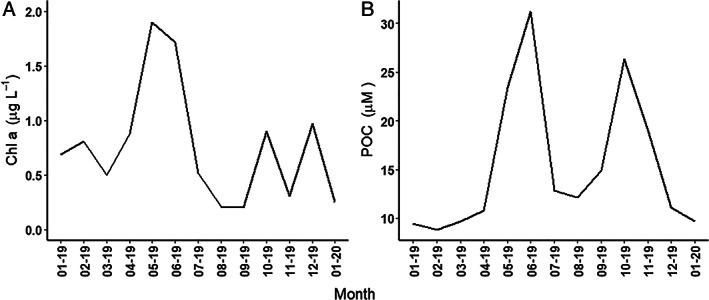
Trends of (A) Chl *a* and (B) POC measured *in situ* from January 2019 to January 2020. Sampling dates are indicated with the format mm‐yy.

PA and FL fractions showed rather similar temporal dynamics (Fig. 1). Selfish activity maxima were measured in June 2019 for all substrate incubations (Fig. 1); winter minima characterized pullulan and xylan incubations, whereas laminarin‐ and chondroitin sulfate‐positive cells showed the lowest abundance between September and November 2019 (Fig. 1). In laminarin incubations, the summer peak was mostly evident for the PA fraction, whereas FL cells showed a less pronounced seasonal variability (Fig. 1). Chondroitin sulfate‐positive cells followed an opposite pattern, with FL cells showing a higher degree of seasonal variability when compared with PA ones (Fig. 1). This temporal pattern appeared to be linked to the overall Chlorophyll *a* and particulate organic carbon (POC) temporal dynamics, both peaking between May and June (Fig. 2, determined as detailed in the study by Manna *et al*., 2021).

The set of FLA‐PS used in the present study includes some of the most abundant or readily hydrolysed polysaccharides in the ocean (Arnosti, 2011; Becker *et al*., 2020), which are likely derived from primary producers (Mühlenbruch *et al*., 2018; Bäumgen *et al*., 2021). The study area is heavily influenced by riverine inputs, representing the major source of inorganic nutrients (Cozzi *et al*., 2020), driving seasonal and interannual changes in both autochthonous and allochthonous OM standing stocks and, consequently, in microbial OM utilization patterns (Celussi and Del Negro, 2012; Manna *et al*., 2021).

Xylan is a structural polysaccharide contained in land plants as well as in macroalgae and phytoplankton cell walls (Beg *et al*., 2001), and its fundamental units (i.e. xylose molecules, Bastawde, 1992) are broadly present in the HMW‐OM pool in the study area, peaking between May and June (Sist and Urbani, 2017). The extensive presence of these molecules, likely due to the influence of terrestrial runoff at our coastal sampling station, matches with maxima observed in FLA‐xylan amended incubations (Fig. 1), establishing a link between HMW‐OM composition and selfish polysaccharide degradation.

In the investigated system, *Synechococcus* spp. dominates as the main photoautotrophic plankton over the nutrient‐depleted summer (Fonda Umani *et al*., 2012). Among the substrates tested, prolonged summer maxima were only observed in chondroitin sulfate incubations (June–August, Fig. 1). *Synechococcus* strains naturally produce chondroitin sulfate (Sarnaik *et al*., 2019), suggesting that these organisms may have represented a natural source of chondroitin sulfate, promoting the selfish uptake of this substrate. We found indeed a significant correlation between total FLA‐chondroitin sulfate‐positive cells and *Synechococcus* abundance (Spearman's rho = 0.94, *P* < 0.001, *n* = 10), linking the observed selfish uptake pattern with *Synechococcus* dynamics, an important nutritional source for heterotrophic microbes in the study area (Manna *et al*., 2021).

We noted that during the investigation period, the selfish uptake of arabinogalactan was not observed. Arabinogalactans are branched polysaccharides composed of arabinose and galactose monomers (Showalter, 2001), also present in brown macroalgae (Hervé *et al*., 2016). Along the easternmost coast of the northern Adriatic Sea, where our sampling site is located, brown macroalgae are broadly present (Orlando‐Bonaca *et al*., 2013), suggesting that arabinogalactan may be an abundant polysaccharide in this area. Arabinose and galactose monosaccharides, the fundamental units of the arabinogalactan molecule, represent a consistent fraction of the dissolved OM pool at the sampling station (7%–11% and 17%–27%, respectively; Sist and Urbani, 2017). Since the signature of external hydrolysis is the production of LMW products (i.e. mono‐ or disaccharides, Vetter *et al*., 1998), we speculate that the bulk of arabinogalactan in the study area is carried out through external hydrolysis (i.e. through cell‐bound or cell‐free exoenzymes), thus reducing the availability of the whole arabinogalactan molecule for selfish microbes. However, this hypothesis remains to be tested by direct measurement of arabinogalactan hydrolysis rates.

**Fig. 3:**
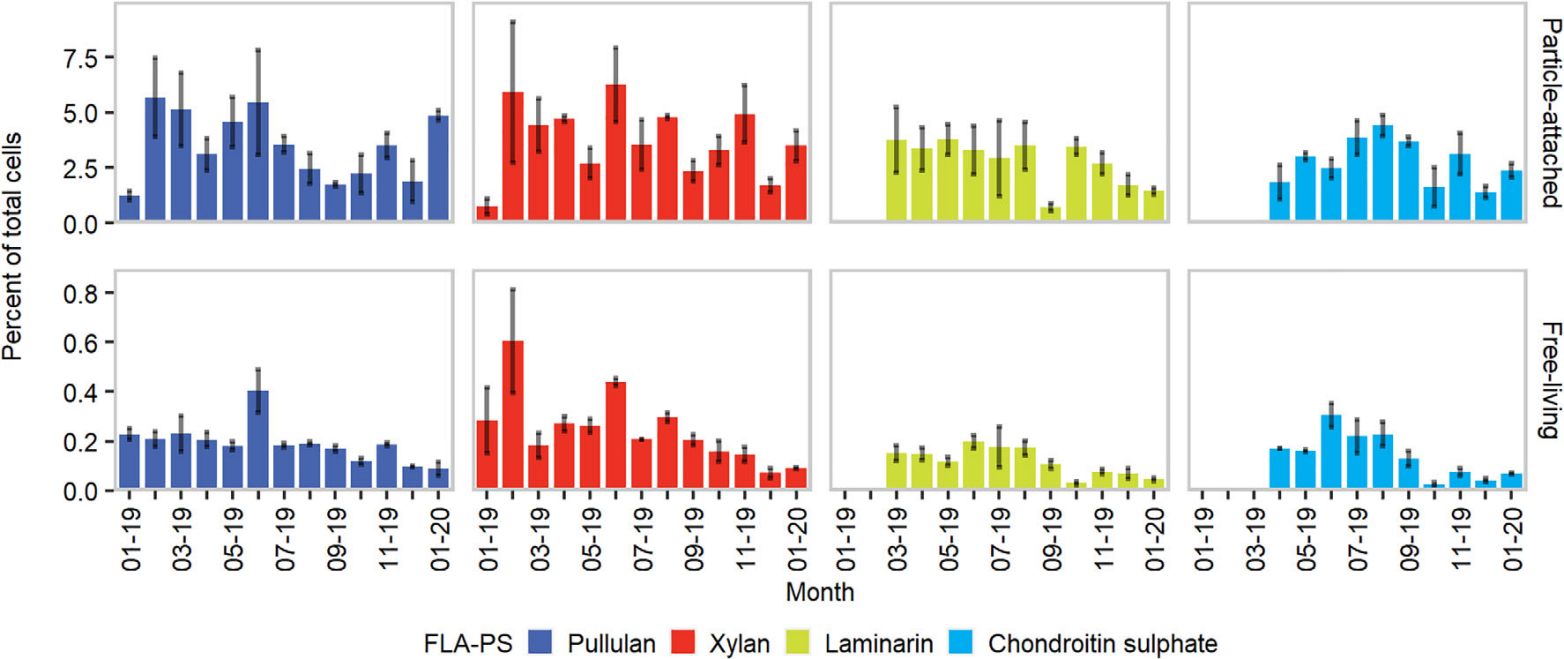
Temporal trends of selfish cells (percentage over total DAPI‐stained cells) in unamended (i.e. without phytodetritus) samples incubated with different FLA‐PS. Error bars depict standard deviations calculated from three experimental replicates. For visualization purposes, *X*‐axis labels are displayed every other month.

In terms of absolute cell abundance, PA and FL substrate‐stained cells showed in general similar dynamics (Fig. 1). However, when compared to the total DAPI‐stained cell counts, the uptake of fluorescent polysaccharides emerged as more widespread among PA microbes (Fig. 3).

Substrate‐stained cells represented between 0.72% ± 0.35% and 6.25% ± 1.68% of the total PA cells in unamended incubations. The highest values were observed in pullulan and xylan incubations (5.67% ± 1.40% and 6.25% ± 1.68%, respectively; Fig. 3), whereas the contribution of laminarin and chondroitin sulfate‐stained cells to the total PA abundance was lower (laminarin: 0.72% ± 0.18%–3.77% ± 0.69%; chondroitin sulfate: 1.39% ± 0.26%–4.42% ± 0.47%; Fig. 2). Gene groups implicated in polysaccharides utilization (e.g. TBDTs and carbohydrate‐active enzymes) are generally more abundant in the PA fraction of a given microbial community (Smith *et al*., 2013; Simon *et al*., 2014; Bunse *et al*., 2021), suggesting that they have the potential to express the metabolic machinery for selfish polysaccharide uptake. Free‐living substrate‐stained cells represented a remarkably lower fraction of the total, generally below 0.5% (Fig. 3). This result was unexpected since the selfish mechanism allows the exploitation of HMW‐OM minimizing the production of hydrolysis by‐products, which, under the current microbial foraging theory, would be an advantageous scenario for FL microbes (Baltar, 2018).

However, the origin of the polysaccharides used in our incubations suggests that microbes closely associated with living and/or detrital POM are likely equipped to exploit these carbon sources, either exclusively in a selfish way (e.g. fucoidan; Reintjes *et al*., 2019) or concurrently with external hydrolysis (e.g. laminarin; Reintjes *et al*., 2020b). These polysaccharides indeed, can be directly produced by phytoplankton, as storage or structural compounds (laminarin and xylan, respectively; Hsieh and Harris, 2019; Becker *et al*., 2020), by fungi as an extracellular coating agent (pullulan, Singh *et al*., 2008), by macroalgae and seagrasses as cell wall components (Hervé *et al*., 2016; Pfeifer *et al*., 2020) or resemble algal‐derived polysaccharides (e.g. chondroitin sulfate; Bhaskar *et al*., 2005; Zhang *et al*., 2008). Furthermore, the selfish uptake strategy appears to be widespread across marine bacterial taxa known to be associated with organic particles (e.g. Flavobacteriaceae, Planctomycetes and Gammaproteobacteria representatives; Reintjes *et al*., 2019; Supporting Information Fig. S2). In the study area, POM concentration is a major forcing of heterotrophic microbial metabolism (Manna *et al*., 2021), indicating that particles represent an important driver in planktonic microbial foraging dynamics. From this perspective, the selfish uptake of specific polysaccharides may represent a substantial advantage in the race for POM exploitation, likely explaining its prevalence among PA microbes (Fig. 3).

### Short‐term dynamics of selfish polysaccharides uptake

**Fig. 4:**
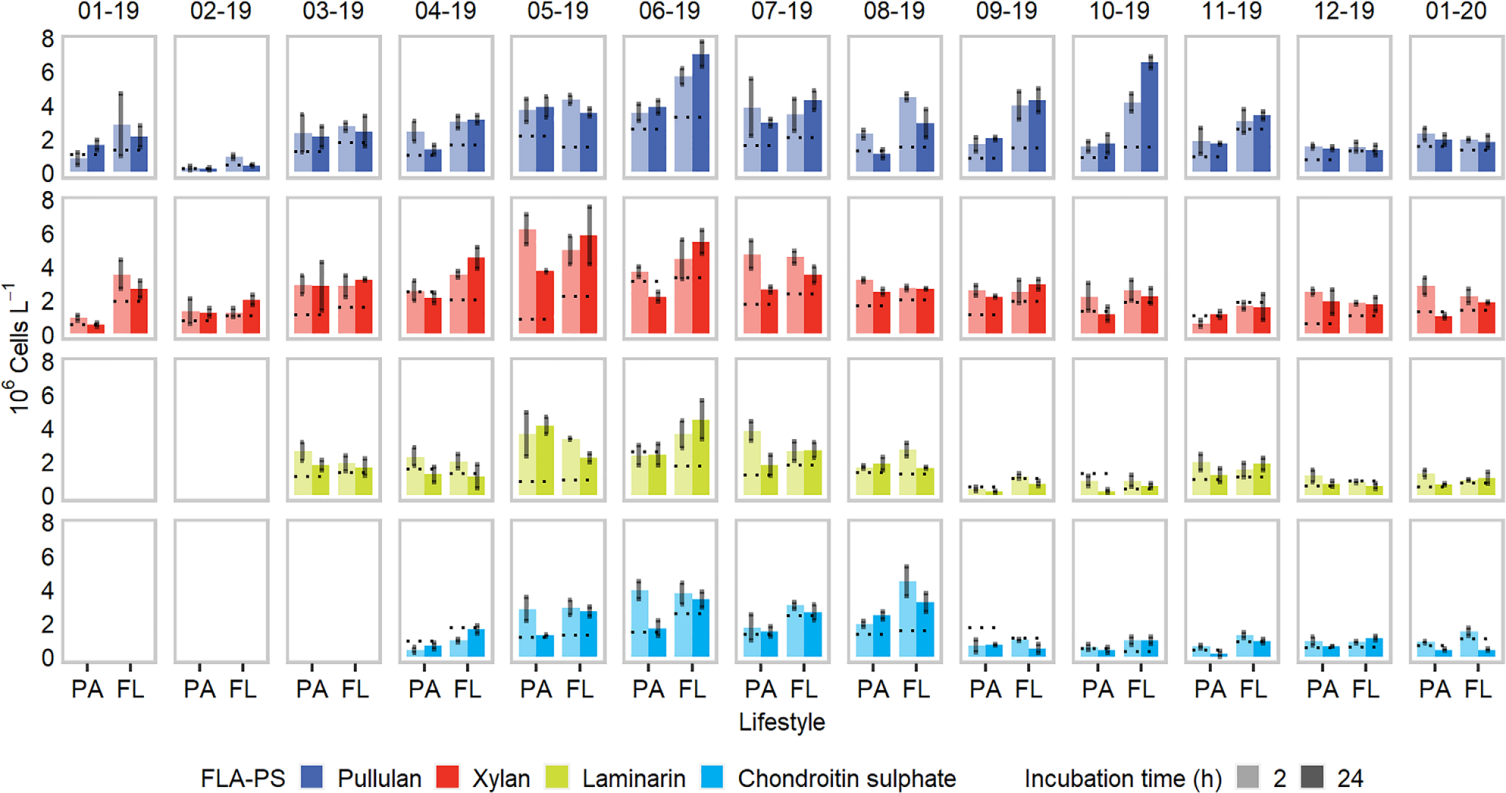
Temporal trends of selfish cell counts in phytodetritus‐amended samples incubated with different FLA‐PS. Error bars depict standard deviations calculated from three experimental replicates. Superimposed dashed lines report values of FLA‐PS positive cells as in Fig. 2 to aid comparison. On the top of each subpanel, the sampling date (mm‐yy format) is present. PA = particle‐attached; FL = free‐living.

Organic particles represent dynamic environments, where the competition for resource access is likely to be strong (Long and Azam, 2001; Bižić‐Ionescu *et al*., 2015). During the particles' colonization and degradation processes, different microbial assemblages develop over time, delineating a successional transition from primary particle degraders to secondary consumers (Datta *et al*., 2016; Bunse *et al*., 2021). The latter are often represented by cheating microbes, benefitting from hydrolysis by‐products released by primary degraders (Datta *et al*., 2016; Koch *et al*., 2019; Baltar *et al*., 2021; Bunse *et al*., 2021). Selfish polysaccharides uptake may represent an effective strategy that primary particle degraders implement to avoid being outcompeted by secondary consumers. However, this behaviour may be difficult to observe in naturally occurring large particles, since they are colonized by climax communities in which individual organisms occupy a defined degradation niche (Datta *et al*., 2016; Bunse *et al*., 2021). To test whether selfish polysaccharides uptake would be a prominent strategy in the early phase of particle colonization, we supplied model phytodetrital particles (prepared as described in the Supporting Information S1) to natural communities, after the removal of large (i.e. >5 μm) particles. In this ‘new colonization’ scenario, we observed that selfish polysaccharide uptake was more common among PA cells, and very few FL cells showed selfish activity at either time point (i.e. 2 h and 24 h; Fig. 4 and Supporting Information Fig. S3), confirming the results observed for unamended natural samples.

Phytodetritus enrichments resulted in a general increase of substrate‐stained cells when compared with unamended incubations (Figs. 3 and 4, respectively). However, selfish cells still represented a minor fraction of the total (i.e. DAPI stained) cells, with higher relative proportions observed in the PA fraction (ranges: PA 2 h, 0.28 ± 0.06%– 6.53 ± 2.83%; FL 2 h, 0.04 ± 0.01%–0.51 ± 0.11%; PA 24 h, 0.05 ± 0.06%–5.90 ± 0.76%; FL 24 h, 0.01 ± 0%–0.54 ± 0.09%; Supporting Information Fig. S3). A paired T‐test (Student, 1908) was applied to detect significant changes in (i) total DAPI‐stained cell and (ii) FLA‐PS‐stained cell abundance after 2 h and 24 h of incubation with detrital particles (details are present in the Supporting Information S1). As in previous FLA‐PS incubation experiments, we expected to observe an increase in selfish cell abundance over the 24 h incubation period (e.g. Reintjes *et al*., 2019). We found instead a significant decrease of selfish cells between 2 h and 24 h, except for pullulan and FL cells in xylan incubations (Table 1). DAPI‐stainable cells significantly increased over the 24 h substrate incubations, except for PA cells amended with FLA‐xylan (Table 1). These results contrast with previous FLA‐PS incubation experiments (Reintjes *et al*., 2019).

With the addition of phytodetrital particles, we aimed to mimic the late stage of a phytoplankton bloom. In similar environmental conditions (i.e. the demise of a phytoplankton bloom, Reintjes *et al*., 2020b), it has been observed that polysaccharide utilization through the selfish mechanism is reduced, while the spectrum and rates of external hydrolysis increase. In this scenario, fast‐growing external hydrolysers may become prevalent in a given microbial community, limiting the flux of HMW molecules processed in a selfish manner (Reintjes, *et al*., 2020b; Arnosti *et al*., 2021). Given this framework, the concurrent decrease of FLA‐PS‐positive cells and the increase of total DAPI‐stained cells observed during our incubation with phytodetrital particles would suggest that, despite a relatively short time frame (i.e. 24 h), selfish cells may have been outcompeted by external degraders, in line with previous, although longer, experiments (Reintjes *et al*., 2020b). However, since the selfish uptake is considered to be particularly effective in presence of highly complex substrates, it is possible that increasing the incubation time, the selfish behaviour among PA‐bacteria would become prevalent in the effort of degrading the refractory OM accumulating over time.

**Table 1:** Summary table of paired T‐test statistics carried out on log‐transformed cell counts in phytodetritus amended incubations.

		DAPI stained				
		Mean ± SD		T‐test statistics		
		2 h	24 h	df	t	*P*
Pullulan	PA	18.51 ± 0.83	18.69 ± 0.74	37	3.27	<0.01
	FL	21.03 ± 0.52	21.25 ± 0.60	37	5.41	<0.001
Xylan	PA	18.55 ± 0.66	18.62 ± 0.76	37	1.37	*ns*
	FL	21.02 ± 0.55	21.25 ± 0.59	37	6.21	<0.001
Laminarin	PA	18.73 ± 0.38	19.01 ± 0.42	32	5.48	<0.001
	FL	21.18 ± 0.25	21.47 ± 0.33	32	5.78	<0.001
Chondroitin sulfate	PA	18.82 ± 0.27	19.07 ± 0.36	29	4.64	<0.001
	FL	21.23 ± 0.24	21.48 ± 0.23	29	7.07	<0.001

		FLA‐PS‐stained				
		Mean ± SD		T‐test statistics		
		2 h	24 h	df	*t*	*P*
Pullulan	PA	14.38 ± 0.78	14.30 ± 0.76	37	−1.01	*ns*
	FL	14.87 ± 0.52	14.80 ± 0.76	37	−1.06	*ns*
Xylan	PA	14.67 ± 0.66	14.37 ± 0.52	37	−3.88	<0.001
	FL	14.80 ± 0.43	14.84 ± 0.46	37	0.656	*ns*
Laminarin	PA	14.29 ± 0.71	13.81 ± 0.97	32	−4.69	<0.001
	FL	14.31 ± 0.58	14.08 ± 0.72	32	−2.86	<0.01
Chondroitin sulfate	PA	13.85 ± 0.81	13.51 ± 0.78	29	−2.96	<0.01
	FL	14.33 ± 0.63	14.10 ± 0.78	29	−2.33	<0.05

Mean and standard deviations (SD) are calculated on log‐transformed DAPI‐ and FLA‐PS‐stained cell counts (ln cells L^−1^).

PA, particle attached; FL, free living; ns, not significant; df, degrees of freedom.

Since the detrital pool used for phytodetritus‐amended incubation was diatom‐based, we expected to observe a high prevalence of laminarin‐stained selfish cells, as laminarin is central in diatoms metabolism (Biersmith and Benner, 1998). Despite this premise, we did not observe a major increase of laminarin selfish uptake when compared to the other substrates tested in the phytodetritus‐amended incubations (Fig. 4 and Supporting Information Fig. S3). Laminarin is an abundant polysaccharide in the marine environment (Becker *et al*., 2020) and thus this substrate is likely targeted not only by selfish uptake but also processed through external hydrolysis, especially when it is more bioavailable, as during the late stages of a diatom bloom (Reintjes *et al*., 2020b).

The outcomes of phytodetrital amendments would suggest that early particles colonizers tend to maximize polysaccharides exploitation via selfish uptake. We found indeed higher numbers of PA selfish cells in early colonization stages, decreasing as the particles, and their associated communities, age. However, the cumulative evidence gathered on the selfish substrate processing mode suggests that there is a gradient of ‘selfishness’ (see Arnosti *et al*., 2021 for a review). Processing polysaccharides through a selfish mechanism may grant exclusive access to specific HMW‐OM components, providing a competitive advantage. Furthermore, selfish processing of certain substrates might still release oligomers (i.e. in the case of ‘sensing enzymes’, *sensu* Thomas *et al*., 2012), which in turn may induce the external hydrolysis of the target compounds (Arnosti, 2004), further enhancing the production of LMW hydrolysate, benefitting the entire community (Smith *et al*. 1992). Therefore, it appears that the selfish uptake mechanism is not solely useful in competing for resource access but plays a key role also in cooperative interactions.

## Conclusions

As the first evidence of selfish polysaccharide uptake in the ocean resulted in an adjustment of the sharing‐cheating model (Allison, 2005; Reintjes *et al*., 2019), the finding of a higher proportion of PA cells performing selfish FLA‐PS uptake challenges the idea that PA microbes behave as sharing entities, releasing external hydrolysis by‐products (i.e. small oligomers and monomers) into the surrounding environment (Smith *et al*., 1992; Simon *et al*., 2002). With this work, we provide evidence that, in temperate coastal waters and for the selected set of polysaccharides, selfish uptake is predominantly carried out by the PA fraction of the heterotrophic microbial community. Furthermore, the incubations with detrital particles highlighted that the selfish strategy is preferentially adopted in the early phase of particles colonization, decreasing in importance as the particles age. However, these results refer to a limited set of molecules, which, although including some of the most abundant or readily degraded polymers in marine systems (Arnosti, 2011; Becker *et al*., 2020), are far from describing OM chemical diversity in the ocean (Riedel and Dittmar, 2014). It is therefore not possible to exclude that in the presence of other polysaccharides, lipids or proteins and/or in other environmental conditions, PA microbes can behave as ‘sharing’ individuals (e.g. Rakoff‐Nahoum *et al*., 2016), or even adopt a combined degradation strategy, coupling external hydrolysis with selfish behaviour (Reintjes *et al*., 2020b). This work underlines the need to extend such investigations, broadening the spectrum of target molecules as well as the breadth of surveyed environments. Coupling this information with insights on microbial lifestyle will be fundamental in future experimental and modelling studies, as microbe‐mediated OM fluxes at the microscale inherently shape the biogeochemistry of the global ocean.

## Supporting information


Appendix S1: Supporting information
