# Supplementary material for: Linking lifestyle and foraging strategies of marine bacteria: selfish behaviour of particle‐attached bacteria in the northern Adriatic Sea
Source: Environ Microbiol Rep. 2022 Mar 31;14(4):549–58. doi: 10.1111/1758-2229.13059 (PMC9546125; doi:10.1111/1758-2229.13059)
Supplement: Supplementary file 1 — Appendix S1: Supporting information [file EMI4-14-549-s001.docx]

***Supplementary Material to:***

Linking lifestyle and foraging strategies of marine bacteria: selfish behaviour of particle-attached bacteria in the Northern Adriatic Sea

Vincenzo Manna^1^, Luca Zoccarato^2^, Elisa Banchi^1^, Carol Arnosti^3^, Hans-Peter Grossart^2, 4^, Mauro Celussi^1^

1: National Institute of Oceanography and Applied Geophysics – OGS, Dept. of Oceanography, Trieste, Italy

2: Leibniz Institute for Freshwater Ecology and Inland Fisheries (IGB), Dept. Experimental Limnology, Zur alten Fischerhuette 2, D-16775 Stechlin, Germany

3: University of North Carolina – Chapel Hill, Dept. of Earth, Marine, and Environmental Sciences, Chapel Hill NC 27599 USA

4: Potsdam University, Inst. For Biochemistry and Biology, Maulbeeralle 2, D-14469 Potsdam, Germany

Corresponding author: Vincenzo Manna, National Institute of Oceanography and Applied Geophysics, Oceanography Section, Via Auguste Piccard 54, 34151, Trieste (TS), Italy

Tel.: +39 0402140737

E-mail: [vmanna@inogs.it](mailto:vmanna@inogs.it)

Running title: Linking lifestyle and foraging modes of marine bacteria

# Supplementary Experimental Procedures

## Sampling and substrate incubations

Seawater was sampled monthly, from January 2019 to January 2020, at a coastal Long-Term Ecological Research station in the Gulf of Trieste (LTER-C1 C1, 45°42’2’’ N, 13°42’36’’ E, north-eastern Adriatic Sea) at ~1 m depth using 5 L Niskin bottles. Subsamples for determination of FLA-PS uptake were then transferred into 1 L, HCl-washed, Nalgene PC bottles, transported to the laboratory into cooler boxes (~15 min) and then processed within 1 h after collection.

FLA-PS uptake was analysed via two approaches, i.e. (i) the evaluation of selfish mechanisms on natural samples and (ii) the study of dynamics of selfish bacteria in phytodetrital particle-amended incubations. FLA-PS were prepared as described in Arnosti (Arnosti, 2003) and Reintjes et al. (Reintjes et al., 2017).

For measurements of FLA-PS uptake by the natural microbial community, 15 mL of surface seawater were added to sterile polypropylene (PP) vials and incubated in the dark at *in situ* temperature with one of the 5 FLA-PS (i.e., pullulan, xylan, laminarin, chondroitin sulphate and arabinogalactan; 5 vials in total) for 2 h. After the incubation, triplicate 5 mL aliquots were filtered onto black PC filters (0.2 µm pore size, Whatman) and stored at -20°C until further processing.

In addition, triplicate 40 mL aliquots were filtered through 5 µm pore-size PC filters (Whatman), to remove large particles. To this fraction (i.e., natural sample <5 µm), *Pseudo-nitzschia* spp. detritus (prepared as described below) was added to simulate decaying-cell abundance during the demise of a phytoplankton bloom at a final concentration of 1 × 10^4^ cells mL^-1^(Cabrini et al., 2012). Tubes were incubated in the dark at *in situ* temperature for 24 hours, to allow for particle colonization. After 24 h, from each colonization vial, 7 mL were transferred into sterile 15 mL PP vials and spiked with one of the 5 FLA-PS (15 vials in total). After 2 and 24 h from the spike, 3 mL from each vial were filtered onto black polycarbonate filters (0.2 µm pore size, Whatman) and stored at -20°C until further analysis.

For each sampling time point, 50 mL of seawater were autoclaved, split into five 10 mL aliquots, each spiked with one of the 5 FLA-PS and incubated at *in situ* temperature for 2 h, serving as killed controls.

Single FLA-PS substrates were added at a concentration of 3.5 µM monomer equivalents to each of the incubated vials and killed controls (Reintjes et al., 2017).

## Phytodetritus preparation

For phytodetritus generation, 12 mL aliquots of a *Pseudo-nitzschia* spp. culture in the stationary growth phase were subjected to 7 cycles of freeze-thawing as previously described (Manna et al., 2020) and stored at -80°C. Before each sampling event, a detritus aliquot was thawed at room temperature and ultrasonicated (10 cycles of 30 s on-off), to detach bacteria from the diatom cells. Since we were interested in the particulate fraction (i.e., detrital diatom cells), ultrasonicated detritus was filtered onto a 5 µm PC membrane, to remove the DOC. Phytodetrital particles were then resuspended in an equal volume of filtered and autoclaved seawater and stored at +4°C until needed (~2 h). Prior to use, diatom detritus was confirmed to be free of bacteria by epifluorescence microscopy, as described below.

## Epifluorescence microscopy

Filtered samples were stained with 4,6-diamidino-2-phenylindole (DAPI, Sigma Aldrich) following the protocol of Porter and Feig (Porter and Feig, 1980) with slight modifications, as reported in Celussi *et al.* (2017). Filters were placed on a drop (50 µL) of DAPI (30 µg mL^-1^ in an autoclaved 3.7% NaCl solution) for 15 min in the dark. The back of the filters was gently dried onto a kimwipe tissue, mounted between layers of immersion oil (Type A, Cargille) and stored at -20 °C until analysis. Total and FLA-PS positive cells were identified and enumerated as free-living or particle-attached at 1000× magnification (Olympus BX60F5) under a UV (BP 330–385 nm, BA 420 nm) and blue (450–480 nm, BA 515 nm) filter set for DAPI and FLA-PS fluorescence, respectively, in at least 15 randomly-selected fields. Cells were identified as substrate-stained if they showed overlapping signals in both DAPI and FLA-PS fields. No FLA-PS-stained cells were observed in the killed controls.

## DNA extraction, 16S library preparation and sequencing

DNA was extracted using the DNeasy PowerWater kit (Qiagen) on 0.2 µm pore-sized PES filters (Supor-200, PALL) after filtration of 1-3L of seawater. The supplier’s instructions were followed with slight modifications to increase the DNA yield and quality (detailed in (Celussi et al., 2018). The concentration of the extracted DNA was assessed with a Qubit Fluorimeter (Thermo Fisher Scientific). For the DNA metabarcoding analysis, the V4-V5 region of the 16S rRNA gene was amplified using 515-Y (5′-GTGYCAGCMGCCGCGGTAA-3′) and 926R (5′-CGYCAATTYMTTTRAGTTT-3′) primers (Parada et al., 2016). Libraries were prepared following the 16S Metagenomic Sequencing Library Preparation protocol and run on an Illumina MiSeq System for a read length of 2 × 250 bp at the genetic and epigenetic ARGO Open Lab Platform, Area Science Park, Trieste, Italy. The 16S amplicon sequences generated for this study can be found in the Sequence Reads Archive (SRA) at NCBI under the BioProject accession number PRJNA767222.

## Data analysis

A paired T-test (Student, 1908) was applied to detect significant changes in (i) total DAPI-stained cells and (ii) FLA-PS-stained cells abundance after 2 h and 24 h of incubation with detrital particles. Data were log-transformed to achieve normality. The test was applied to both PA and FL fractions.

Bioinformatic analyses were performed with QIIME2 2020.6 (Bolyen et al., 2019). Raw sequences were quality filtered and denoised with DADA2 (Callahan et al., 2016). Taxonomy was assigned to amplicon sequence variants (ASVs) using the sklearn naïve Bayesian taxonomy classifier (Bokulich et al., 2018) against the Silva 138 (99%) reference database with 7-level taxonomy (Quast et al., 2013).

ASVs relative abundance was multiplied with prokaryotic abundance measured by flow cytometry (as detailed in Manna et al., 2021) to infer their absolute abundance (Alonso-Sáez et al., 2015). These data were than collated with FLA-PS-stained total cells abundance in each substrate incubation. This analysis was used to infer the selfish potential of the sampled natural communities. Only correlations showing Spearman’s rho≥0.7 were retained for subsequent visualization. All the above tests were considered significant at *p*<0.05, after correction for false discovery rate (fdr; Benjamini and Hochberg, 1995), when appropriate. Data manipulation, analysis and visualization were conducted in the R environment (R Core Team, 2019), using the packages tidyverse v 1.3.0, phyloseq v 1.28.0, pheatmap v 1.0.12, ggplot2 v 3.3.3 (McMurdie and Holmes, 2013; Wickham, 2016; Kolde, 2019; Wickham et al., 2019).

# Supplementary Figures


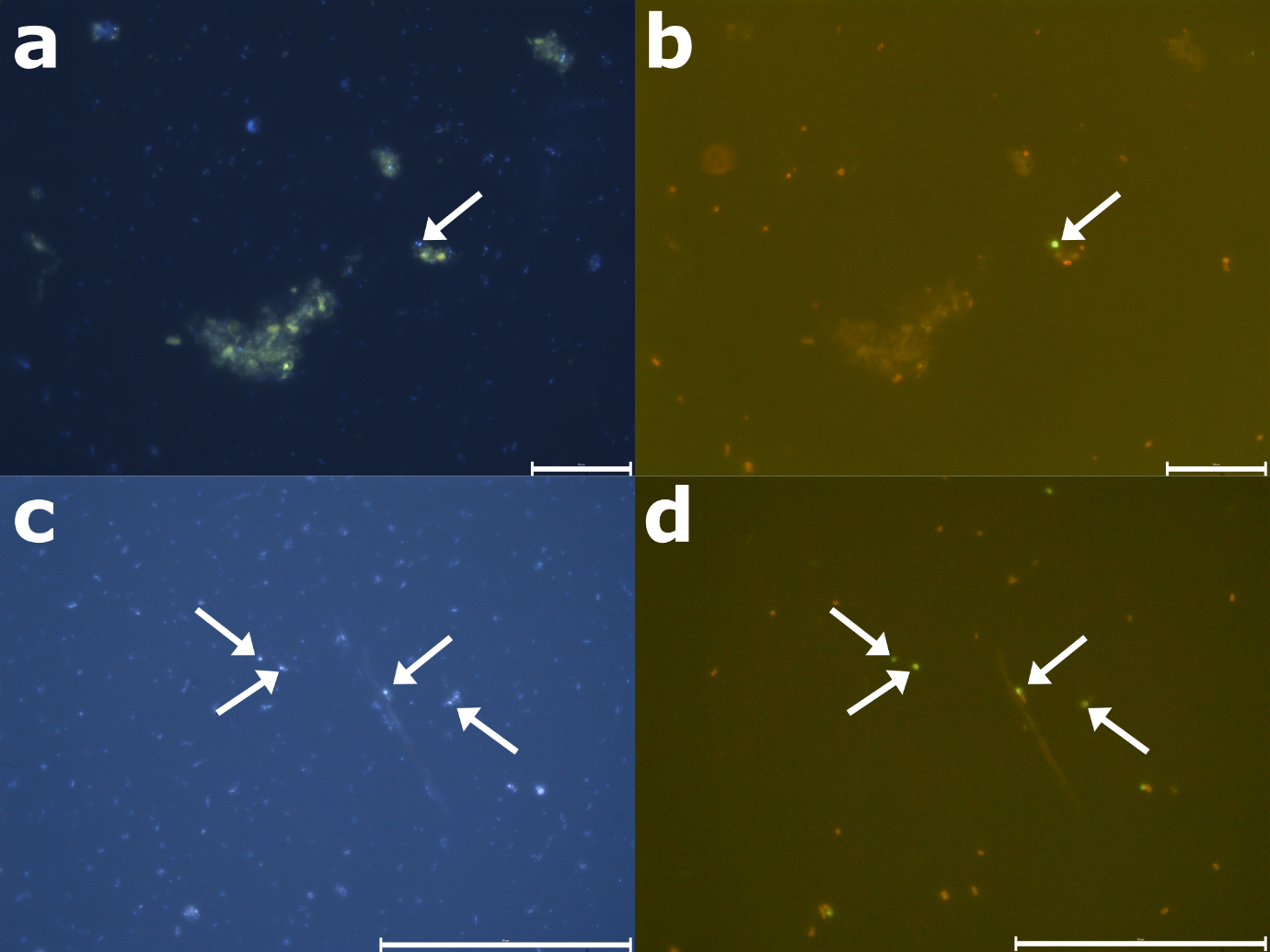


**Supplementary Figure 1** Representative epifluorescence micrographs of cells stained by (**a**, **b**) DAPI (blue), (**c**, **d**) FLA-pullulan (green). FLA-PS positive cells are highlighted by arrows in DAPI and FLA-PS fields. **a** and **b** micrographs represent natural samples amended with FLA-pullulan; a FLA-pullulan-positive cell attached to an amorphous aggregate is visible. **c** and **d** represent FLA-pullulan incubation amended with phytodetrital particles; free-living FLA-pullulan-positive cells are visible as well as one positive cell attached to a phytodetrital particle. Samples from June 2019 were used to shoot these micrographs. Scale bars length: **a** and **b**=20µm; **c** and **d**=50µm.


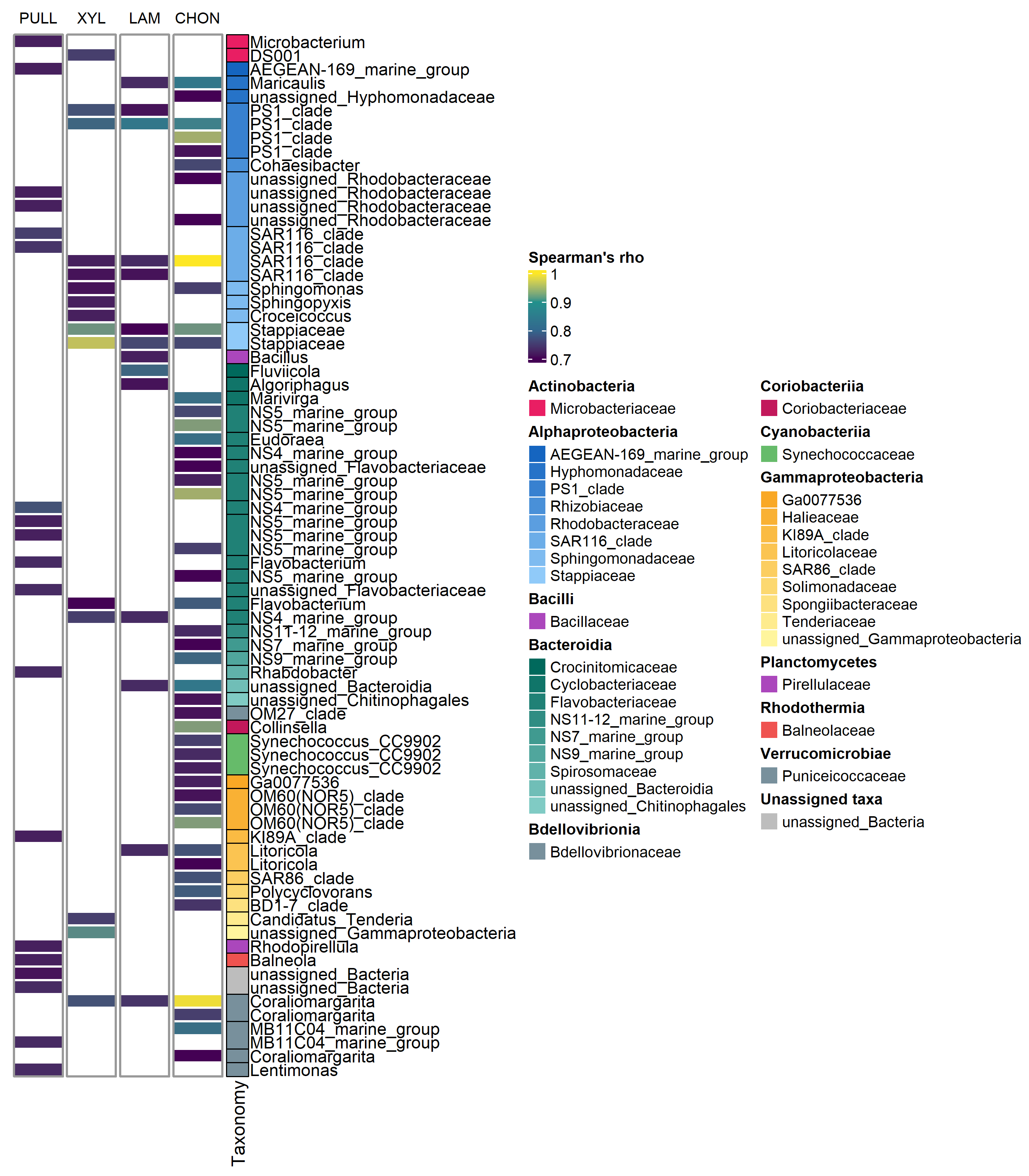


**Supplementary Figure 2** Spearman’s Rank-Order correlations between ASVs absolute abundance (inferred multiplying ASVs relative abundance with flow cytometry counts of total prokaryotes) and absolute abundance of selfish cells in each of the substrate incubations (PULL=pullulan, XYL=xylan, LAM=laminarin, CHON=chondroitin sulphate). Only significant [p(fdr)<0.05] and positive (Spearman’s rho≥0.70) are displayed. Insignificant correlations are left blank. Top legend maps to correlation coefficient; bottom legends maps to ASVs’ higher rank taxonomy. Among the set of taxa whose absolute abundance significantly correlated with the selfish cell counts, there were representatives previously identified as selfish degraders. Members of the Bacteroidia order showed the highest number of significant hits, in accordance with their known ability to selfishly uptake labelled polysaccharides (Reintjes et al., 2017). Verrucomicrobia and Flavobacteriaceae members showed strong relationship with chondroitin selfish uptake, as previously reported (Reintjes et al., 2017). In these incubations, a correlation was also found with the absolute abundance of Synechococcus ASVs; its ability to produce chondroitin sulphate may have represented a natural source of this polysaccharide, promoting the selfish uptake of this substrate. Alphaproteobacteria were mostly linked to xylan and chondroitin-stained cell abundance. These links between natural communities and selfish cell counts corroborate previous findings on the identity of selfish microbes, opening new questions on the role of specific taxa as selfish degraders.


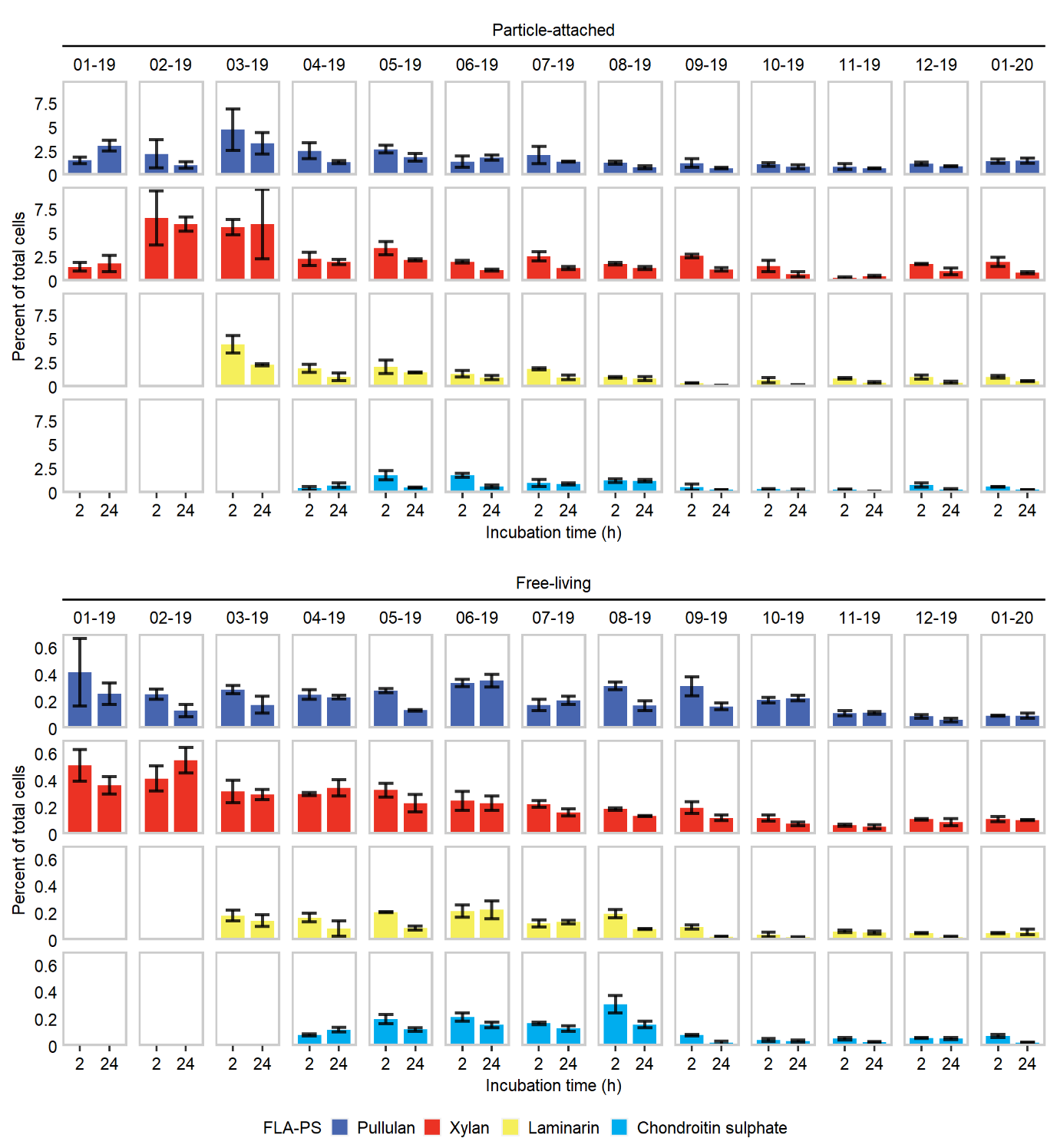


**Supplementary Figure 3** Temporal trends of selfish cells percentage relative to total DAPI-stained cells in phytodetritus-amended samples incubated with different FLA-PS. Error bars depict standard deviations calculated from three experimental replicates. On the top of each subpanel, the sampling date (mm-yy format) is present. Note that Y-axes scales differ between particle-attached and free-living panels.

## Supplementary References

Alonso-Sáez, L., Díaz-Pérez, L., Morán, X. A. G. (2015). The hidden seasonality of the rare biosphere in coastal marine bacterioplankton. Environ. Microbiol. 17, 3766:3780. <https://doi.org/10.1111/1462-2920.12801>

Arnosti, C. (2003). Fluorescent derivatization of polysaccharides and carbohydrate-containing biopolymers for measurement of enzyme activities in complex media. *J. Chromatogr. B. Analyt. Technol. Biomed. Life Sci.* 793, 181–191.

Benjamini, Y., and Hochberg, Y. (1995). Controlling the False Discovery Rate: A Practical and Powerful Approach to Multiple Testing. *J. R. Stat. Soc. Ser. B* 57, 289–300. doi:10.1111/j.2517-6161.1995.tb02031.x.

Bokulich, N. A., Kaehler, B. D., Rideout, J. R., Dillon, M., Bolyen, E., Knight, R., et al. (2018). Optimizing taxonomic classification of marker-gene amplicon sequences with QIIME 2’s q2-feature-classifier plugin. *Microbiome* 6, 90. doi:10.1186/s40168-018-0470-z.

Bolyen, E., Rideout, J. R., Dillon, M. R., Bokulich, N. A., Abnet, C. C., Al-Ghalith, G. A., et al. (2019). Reproducible, interactive, scalable and extensible microbiome data science using QIIME 2. *Nat. Biotechnol.* 37, 852–857. doi:10.1038/s41587-019-0209-9.

Cabrini, M., Fornasaro, D., Cossarini, G., Lipizer, M., and Virgilio, D. (2012). Phytoplankton temporal changes in a coastal northern Adriatic site during the last 25 years. *Estuar. Coast. Shelf Sci.* 115, 113–124. doi:10.1016/j.ecss.2012.07.007.

Callahan, B. J., McMurdie, P. J., Rosen, M. J., Han, A. W., Johnson, A. J. A., and Holmes, S. P. (2016). DADA2: High-resolution sample inference from Illumina amplicon data. *Nat. Methods* 13, 581–583. doi:10.1038/nmeth.3869.

Celussi, M., Malfatti, F., Annalisa, F., Gazeau, F., Giannakourou, A., Pitta, P., et al. (2017). Ocean acidification effect on prokaryotic metabolism tested in two diverse trophic regimes in the Mediterranean Sea. *Estuar. Coast. Shelf Sci.* 186, 125–138. doi:https://doi.org/10.1016/j.ecss.2015.08.015.

Celussi, M., Quero, G. M., Zoccarato, L., Franzo, A., Corinaldesi, C., Rastelli, E., et al. (2018). Planktonic prokaryote and protist communities in a submarine canyon system in the Ligurian Sea (NW Mediterranean). *Prog. Oceanogr.* 168, 210–221. doi:10.1016/j.pocean.2018.10.002.

Kolde, R. (2019). pheatmap: Pretty Heatmaps. Available at: https://cran.r-project.org/package=pheatmap.

Manna, V., Malfatti, F., Banchi, E., Cerino, F., De Pascale, F., Franzo, A., et al. (2020). Prokaryotic Response to Phytodetritus-Derived Organic Material in Epi- and Mesopelagic Antarctic Waters. *Front. Microbiol.* 11, 1242. doi:10.3389/fmicb.2020.01242.

Manna, V., De Vittor, C., Giani, M., Del Negro, P., and Celussi, M. (2021) Long-term patterns and drivers of microbial organic matter utilization in the northernmost basin of the Mediterranean Sea. Mar Environ Res 164: 105245. https://doi.org/10.1016/j.marenvres.2020.105245

McMurdie, P. J., and Holmes, S. (2013). phyloseq: An R package for reproducible interactive analysis and graphics of microbiome census data. *PLoS One* 8, e61217. Available at: http://dx.plos.org/10.1371/journal.pone.0061217.

Parada, A. E., Needham, D. M., and Fuhrman, J. A. (2016). Every base matters: assessing small subunit rRNA primers for marine microbiomes with mock communities, time series and global field samples. *Environ. Microbiol.* 18, 1403–1414. doi:https://doi.org/10.1111/1462-2920.13023.

Porter, K. G., and Feig, Y. S. (1980). The use of DAPI for identifying and counting aquatic microflora1. *Limnol. Oceanogr.* 25, 943–948. doi:10.4319/lo.1980.25.5.0943.

Quast, C., Pruesse, E., Yilmaz, P., Gerken, J., Schweer, T., Yarza, P., et al. (2013). The SILVA ribosomal RNA gene database project: Improved data processing and web-based tools. *Nucleic Acids Res.* 41, D590–D596. doi:10.1093/nar/gks1219.

R Core Team (2019). *R: A Language and Environment for Statistical Computing*. Vienna, Austria: R Foundation for Statistical Computing Available at: https://www.r-project.org/.

Reintjes, G., Arnosti, C., Fuchs, B. M., and Amann, R. (2017). An alternative polysaccharide uptake mechanism of marine bacteria. *ISME J.* 11, 1640–1650. doi:10.1038/ismej.2017.26.

Student (1908). The Probable Error of a Mean. *Biometrika* 6, 1–25. doi:10.2307/2331554.

Wickham, H. (2016). *ggplot2: Elegant Graphics for Data Analysis*. Springer-Verlag New York Available at: https://ggplot2.tidyverse.org.

Wickham, H., Averick, M., Bryan, J., Chang, W., McGowan, L. D., François, R., et al. (2019). Welcome to the {tidyverse}. *J. Open Source Softw.* 4, 1686. doi:10.21105/joss.01686.
